# Efficacy and safety of monoclonal antibodies against respiratory syncytial virus disease in premature infants: a systematic review and network meta-analysis

**DOI:** 10.3389/fped.2026.1775795

**Published:** 2026-07-02

**Authors:** Shunli Liu, Yan Wang, Yang Cao, Huiling Song, Zhiqing Tian, Xue Li, Lan Huang

**Affiliations:** 1 Department of Emergency, West China Second University Hospital, Sichuan University, Chengdu, China; 2 Key Laboratory of Birth Defects and Related Diseases of Women and Children (Sichuan University), Ministry of Education, Chengdu, China; 3 Department of Pediatrics, West China Second University Hospital, Sichuan University, Chengdu, China

**Keywords:** efficacy, meta-analysis, monoclonal antibodies, premature infants, RSV infection, safety

## Abstract

**Objective:**

Respiratory syncytial virus (RSV) is a leading cause of lower respiratory tract infection (LRTI) in infants and young children, which may progress to bronchiolitis, pneumonia, and other severe respiratory complications. This study aimed to evaluate the efficacy and safety of monoclonal antibodies (mAbs) against the RSV disease in premature infants.

**Methods:**

A comprehensive literature search was conducted across the PubMed, Cochrane Library, and Embase databases from inception through December 2025. Statistical analyses were performed using STATA software (version 15.0). Effect estimates were expressed as relative risk (RR) with corresponding 95% confidence intervals, and treatment rankings were evaluated using the surface under the cumulative ranking curve (SUCRA) probability.

**Results:**

This Network Meta-analysis (NMA) included 7 randomized controlled trials involving 4 drugs and 11319 infants. Compared with the placebo, nirsevimab, motavizumab, and palivizumab have shown beneficial effects at reducing RSV-related hospitalization rates (RR, nirsevimab: 0.20, 95% CI: 0.09-0.45; motavizumab: 0.32 95% CI: 0.19-0.53; palivizumab: 0.43; 95% CI: 0.30-0.61) and Intensive Care Unit (ICU) admissions due to RSV infections (nirsevimab: 0.09, 95% CI: 0.01-0.87; motavizumab: 0.23, 95% CI: 0.08-0.65; palivizumab: 0.43, 95% CI: 0.21-0.90) in preterm infants. Nirsevimab was most likely to exert the best therapeutic effects, with the highest (SUCRA) values of 94.4% for RSV-related hospitalization rates and 89.1% for ICU admissions. No significant differences were found in mechanical ventilation use, deaths related to RSV Infections and drug-related adverse events.

**Conclusion:**

Nirsevimab, motavizumab, and palivizumab all reduced RSV-related hospitalizations and ICU admissions in preterm infants, with nirsevimab showing the greatest effect. However, further studies are needed to confirm these findings.

**Systematic Review Registration:**

https://www.crd.york.ac.uk/PROSPERO/view/CRD42024583222, identifier CRD42024583222.

## 1 Introduction

Respiratory Syncytial Virus (RSV) is a highly contagious respiratory-tropic virus that infects individuals of all age groups (1). Although RSV infection typically causes only mild upper respiratory symptoms in healthy adults, it can lead to severe clinical outcomes in high-risk populations, including infants, young children, the elderly, and immunocompromised individuals (1, 2). Globally, RSV is the leading cause of severe lower respiratory tract infections (LRTIs), such as bronchiolitis and pneumonia, in children under five years of age, and it also contributes substantially to respiratory-related hospitalizations among older adults (2, 3). Severe RSV infection imposes considerable healthcare utilization and socioeconomic burdens worldwide and increases the risk of long-term respiratory morbidity in children (3, 4).

In 2019, an estimated 33 million episodes of RSV-associated lower respiratory tract infections occurred in children under 5 years of age, with 95% of these cases concentrated in low- and middle-income countries (5, 6). Approximately 68.8% of infants acquire RSV infection during their first year of life, rising to 82.6% by age 2 years; among those infected, up to 35.4% develop lower respiratory tract complications following the initial infection (7). In the absence of virus-specific antiviral therapy, clinical management of RSV infection relies predominantly on supportive care. Preterm infants and those with underlying conditions—including congenital heart disease (CHD), chronic lung disease (CLD), Down syndrome, neuromuscular disorders, and congenital or inherited airway anatomical abnormalities—are at markedly elevated risk of severe RSV infection (8–13). Accordingly, combating RSV infection in high-risk infants is of critical importance.

Over the past two decades, monoclonal antibodies (mAbs), including palivizumab, nirsevimab, and motavizumab, have been shown to be effective in reducing RSV infection in infants and elderly individuals (14–17). These studies have provided critical evidence to inform public health strategies and guide the development of vaccination policies. Although several systematic reviews have recently examined the use of mAbs against RSV infection, most have focused on children broadly, without distinguishing between preterm and term infants (18). Given that preterm infants are at particularly high risk for RSV infection, evaluating the efficacy and safety of mAbs against RSV disease specifically in this population is of substantial clinical importance. Therefore, we conducted a systematic review and network meta-analysis to evaluate the comparative efficacy and safety of various monoclonal antibodies against RSV infection in premature infants.

## 2 Methods

### 2.1 Registration

The protocol for this systematic review and network meta-analysis has been registered in PROSPERO (registration number: CRD42024583222). This study was conducted and the results were reported following the Preferred Reporting Items for Systematic Reviews and Meta-Analyses (PRISMA) guidelines (19).

### 2.2 Search strategy

To identify eligible studies, we searched the PubMed, Cochrane Library, and Embase databases from inception through December 2025, using keywords including monoclonal antibodies, nirsevimab, motavizumab, palivizumab, suptavumab, clesrovimab, premature infants, and respiratory syncytial virus (Supplementary Data S1). The reference lists of relevant studies were also manually screened to identify additional eligible studies. Duplicates were first removed automatically using EndNote, followed by manual screening of titles and abstracts for further deduplication. Two reviewers independently screened the search results. Full texts of potentially eligible trials were retrieved and independently assessed against the predefined eligibility criteria. Any discrepancies between reviewers were resolved through discussion.

### 2.3 Selection criteria

The inclusion criteria were: (1) participants were premature infants with a gestational age of less than 37 weeks, with or without underlying diseases such as CLD, CHD, among others; (2) the intervention was mAb, with no restrictions on intervention time, frequency, or duration; (3) the control group included patients treated with placebo or no intervention; (4) outcome measures included the number of hospitalizations due to RSV infection, lower respiratory tract infections requiring medical attention for RSV, the number of ICU admissions for RSV infections, the number of individuals requiring mechanical ventilation for RSV infections, RSV-related deaths, and the occurrence of adverse events in the RSV vs. placebo groups; (5) study designs included RCTs.

The exclusion criteria were: (1) studies enrolling non-premature infants; (2) drug administration not via the intramuscular or intravenous injection; (3) non-English language articles; (4) inability to obtain the original text; (5) duplicate publications; (6) non-RCT trials, letters to the editor, reports, reviews, conference abstracts, or study protocols; and (7) animal studies.

### 2.4 Data extraction

Two researchers independently collected all the data using a data extraction form. Any disagreements were resolved through discussion. The primary information included the author, year, sample size, follow-up time, participant characteristics, intervention details, and outcomes.

### 2.5 Quality assessment

Two researchers conducted quality assessments. The disagreements were resolved through consensus. The methodological quality of the included studies was evaluated using the Cochrane Risk of Bias (RoB) tool. Funnel plots were used to assess publication bias when 10 or more studies were included in the meta-analysis. We used the CINeMA (Confidence In Network Meta-Analysis) framework to assess the certainty of evidence across six key domains: within-study, reporting, indirectness, imprecision, heterogeneity, and inconsistency (20).

### 2.6 Data synthesis

Statistical analyses were performed using evaluation management software Stata15.0 (StataCorp LP, USA). Relevant packages and commands including network meta, mvmeta, ifplot, sidesplit, netleague, intervalplot, network rank and sucra prob were applied. A random-effects model network meta-analysis was conducted using a frequentist framework with a consistency model. League tables were used to present RR values and 95% CI for all interventions. The SUCRA values from the cumulative probability plot were used to visually rank the likelihood of each intervention being the most effective.

## 3 Results

### 3.1 Study Selection

**Figure 1:**
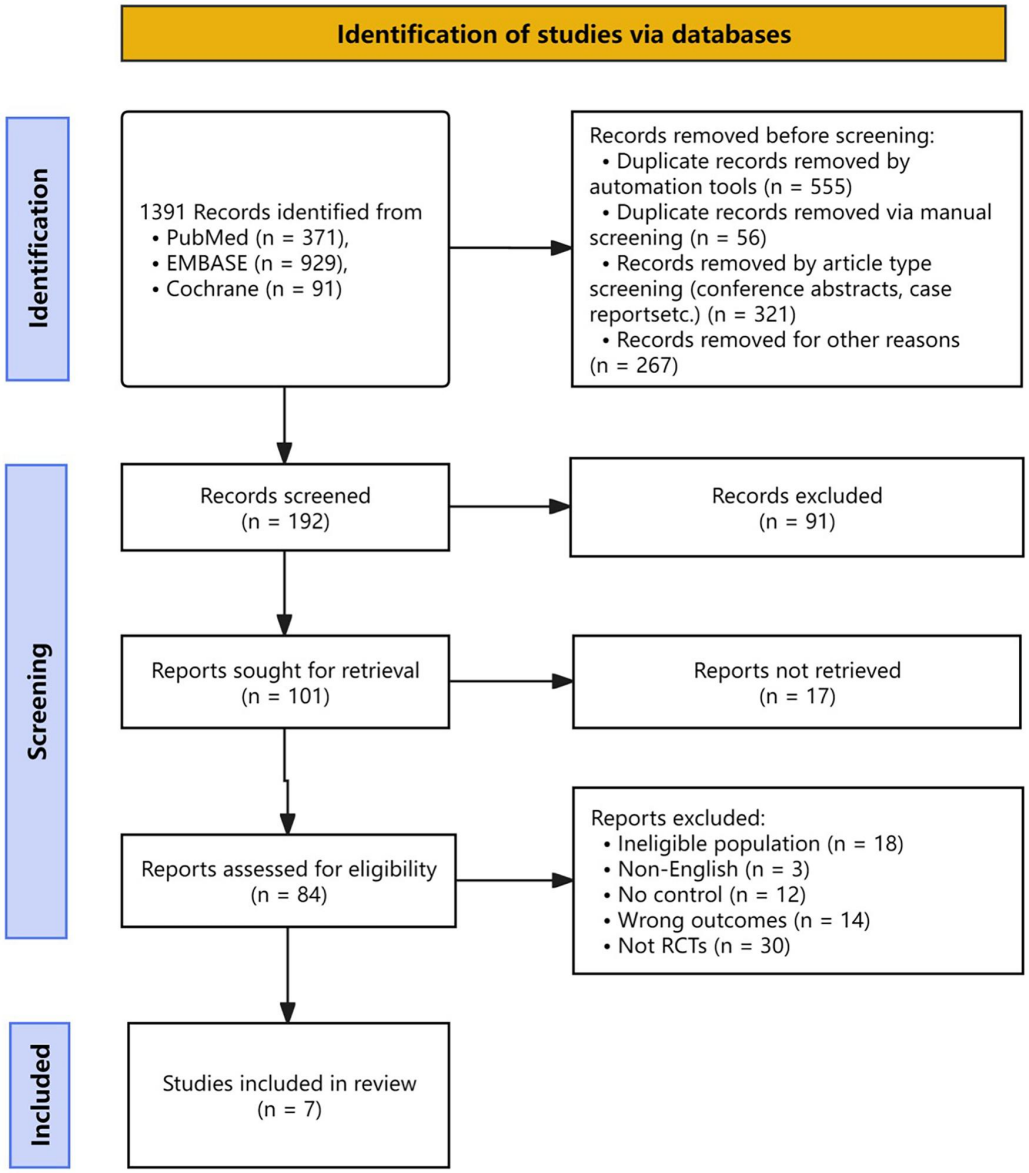
PRISMA flow diagram of included studies evaluating the efficacy and safety of monoclonal antibodies against RSV disease in premature infants.

A total of 1,391 relevant articles were retrieved through a combined manual and computer-based search from the PubMed, Cochrane Library, and EMBASE databases, spanning from inception to December 2025. After screening and exclusion, 7 articles were included (21–27) (Figure 1). Supplementary data S2 presents the studies excluded at the final screening stage, along with the specific reasons for their exclusion.

### 3.2 Characteristics of included studies

This network meta-analysis included seven randomized controlled trials (RCTs): three comparing palivizumab with placebo, two comparing nirsevimab with placebo, one comparing suptavumab with placebo, and one comparing palivizumab with Motavizumab. A total of 11,319 infants were enrolled across these studies (58.7% male, 41.3% female). Eligible infants were preterm with a gestational age of ≤35 weeks; three studies additionally included infants with bronchopulmonary dysplasia (BPD). Among all enrolled infants, 9,699 were treated with monoclonal antibodies and 1,620 with placebo. The duration follow-up period ranged from 150 days to 1 year (Table 1).

**Table 1:** Characteristics of included studies evaluating the efficacy and safety of monoclonal antibodies against RSV disease in premature infants.

**Author/Ref.**	**Year/country**	**Population Sample**	**Group (EG/CG)**	**No. (EG/CG)**	**Sex (Male, %)(EG/CG)**	**Median age(EG/CG)**	**Gestational age, mean (SD),wk(EG/CG)**	**Follow duration**	**Intervention (dose)/comparison**
The IMpact-RSV Study Group (21)	1998/US	Preterm infants (GA < 35wk) with BPD	Palivizumab/Placebo	1,002/500	56.9/56.8	5.7 ± 0.15 m/6 ± 0.21 m	29 ± 0.1/29 ± 0.14	150d	15 mg/kg/dose/monthly, 5 doses/Placebo
Griffin, (22)	2020/US	Healthy preterm infants (GA 29wk-34 + 6wk)	Nirsevimab/Placebo	969/484	51.7/53.7	3.29 ± 2.22 m/3.28 ± 2.31 m	32.7 ± 1.4/32.7 ± 1.5	150d	1 dose 50 mg/NS
Maarten O. Blanken (23)	2013/UK	Healthy preterm infants (GA 33–35wk)	Palivizumab/Placebo	214/215	58/44	NA	34 ± 3/34 ± 3	1y	2–5 doses of 15 mg/kg/dose/monthly/NS
Simões, (24)	2021/Netherlands	Healthy preterm infants (GA < 36wk)	Suptavumab/Placebo	766/383	54.2/51.4	12.67 w/13.00 ± 6.903wk	NA	150–237d	1 or 2 doses,30 mg/Placebo
Carbonell-Estrany, (25)	2010/US	Preterm infants (GA ≤ 35wk) with BPD	Motavizumab/Palivizumab	3,329/3,306	54.6/54.8	3.99 ± 3.75 m/3.98 ± 3.78 m	31.1 ± 3.1/31.1 ± 3.1	150d	15 mg/kg/dose/monthly, 5 doses/Placebo
Subramanian (26)	1998/US	Preterm infants (GA < 35wk) with BPD	Palivizumab/Placebo	42/20	NA	7.6 ± 1.47 m/5 ± 0.83 m	NA	150d	3,10,15 mg/kg/dose/monthly, 5doses/Placebo
Domachowske (27)	2018/US	Healthy preterm infants (32–34 + 6wk)	Nirsevimab/Placebo	71/18	40.8/38.9	6.50 ± 2.64 m/6.95 ± 2.63 m	33.1 ± 0.8/33.1 ± 0.6	1y	10 mg/25 mg/50 mg, 1 dose/NS

BPD, bronchopulmonary dysplasia; d, day; EG/CG, experimental group/control group; GA, Gestational Age; m, month; NA, not available; No, number; NS, normal saline; SD, Standard deviation; US, United States; UK, United Kingdom; wk, week; y, year; d, day.

### 3.3 Efficacy and safety of mAbs against RSV disease in premature infants

**Figure 2:**
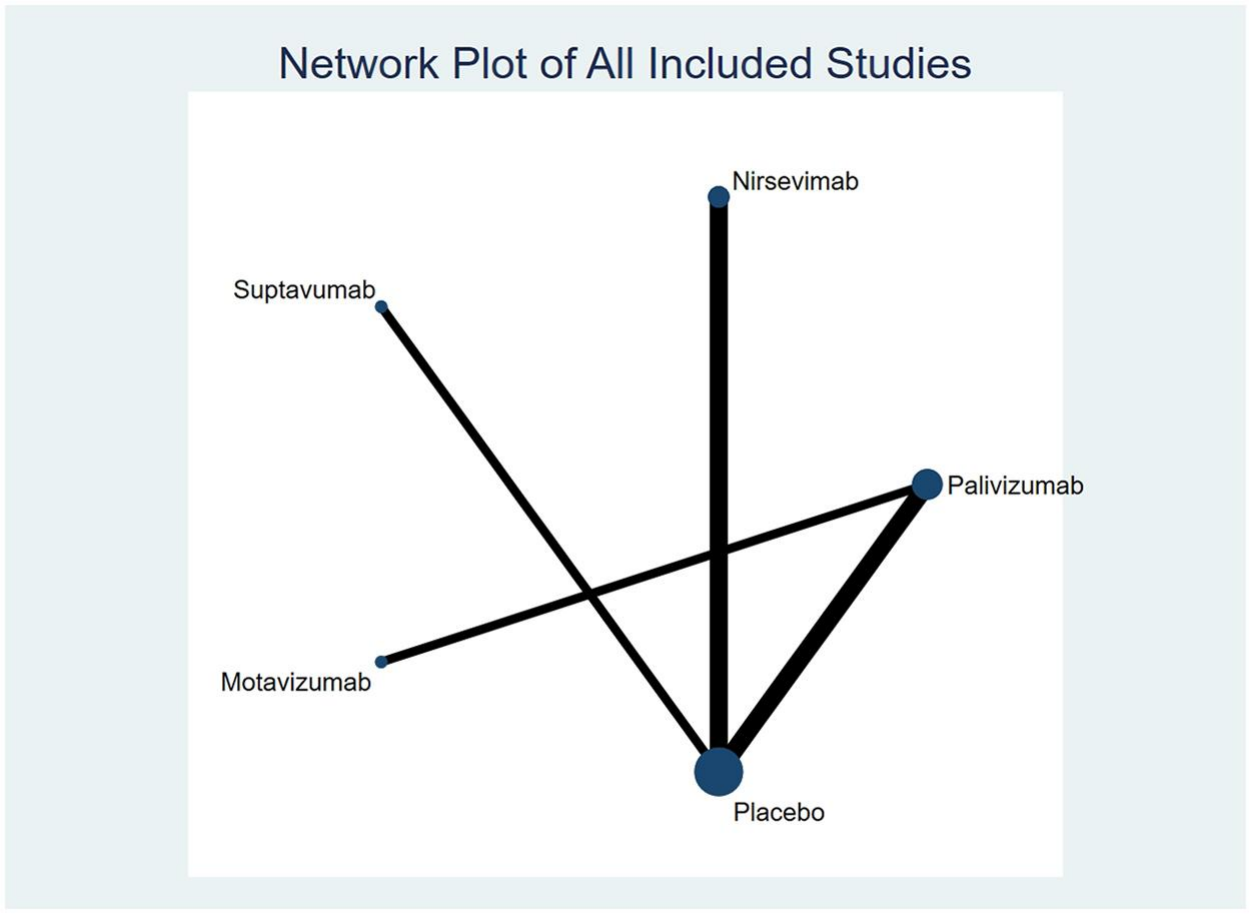
Network plot of included studies evaluating monoclonal antibodies against RSV disease in premature infants. The nodes represent competing interventions, edges represent direct comparisons, and node size reflects the number of participants.

The network diagrams for the seven included RCTs (21–27) are presented in (Figure 2**)**; overall, palivizumab was the most frequently evaluated intervention. We assessed between-study heterogeneity across trials for each comparison. Under the random-effects model, the between-study variance was *τ*^2^ = 4.82 × 10⁻^13^ (between-study standard deviation *τ* = 6.94 × 10⁻^7^), indicating negligible between-study heterogeneity. Owing to the limited number of included studies (only seven), formal assessment of publication bias was not performed. Only the RoB tool was used to assess the quality of the seven eligible studies. Of the seven studies, six were assessed as having a low risk of bias, while one was rated as having some concerns due to selective reporting (Supplementary Data S3, sTable S1). The network exhibited a star-shaped structure without closed loops; therefore, global inconsistency assessment could not be performed. In the absence of closed loops, inconsistency could not be directly estimated for any comparison. Following evidence grading using CINeMA, most pairwise comparisons were rated as having low or very low confidence in the effect estimates (Table 2).

**Table 2:** Evaluation of the quality of the included research literature by using CINeMA.

**Comparison**	**Number of studies**	**Within-study bias**	**Reporting bias**	**Indirectness**	**Imprecision**	**Heterogeneity**	**Incoherence**	**Confidence rating**
Motavizumab:Palivizumab	1	Some concerns	Low risk	No concerns	Some concerns	Some concerns	Major concerns	Very low
Nirsevimab:Placebo	2	No concerns	Low risk	No concerns	No concerns	Some concerns	Major concerns	Very low
Palivizumab:Placebo	3	No concerns	Low risk	No concerns	No concerns	No concerns	Major concerns	Low
Placebo:Suptavumab	1	No concerns	Low risk	No concerns	Major concerns	No concerns	Major concerns	Very low
Motavizumab:Nirsevimab	0	No concerns	Low risk	No concerns	Major concerns	No concerns	Major concerns	Very low
Motavizumab:Placebo	0	Some concerns	Low risk	No concerns	No concerns	No concerns	Major concerns	Very low
Motavizumab:Suptavumab	0	No concerns	Low risk	No concerns	No concerns	Major concerns	Major concerns	Very low
Nirsevimab:Palivizumab	0	No concerns	Low risk	No concerns	Some concerns	Some concerns	Major concerns	Very low
Nirsevimab:Suptavumab	0	No concerns	Low risk	No concerns	No concerns	Major concerns	Major concerns	Very low
Palivizumab:Suptavumab	0	No concerns	Low risk	No concerns	No concerns	Major concerns	Major concerns	Very low

#### 3.3.1 RSV-related hospitalizations

All seven studies (21–27) reported RSV-related hospitalizations (mAb group *n* = 9,699; placebo group *n* = 1,620). Compared with placebo, palivizumab had a RR of 0.43 (95% CI, 0.30–0.61), nirsevimab 0.20 (95% CI, 0.09–0.45), and motavizumab 0.32 (95% CI, 0.19–0.53). All three agents were statistically superior to placebo. When compared with suptavumab, the corresponding RRs for palivizumab, nirsevimab and motavizumab were 0.41 (95% CI, 0.20–0.87), 0.19 (95% CI, 0.07–0.54) and 0.31 (95% CI, 0.13–0.70), respectively, indicating significant superiority as well.

**Figure 3:**
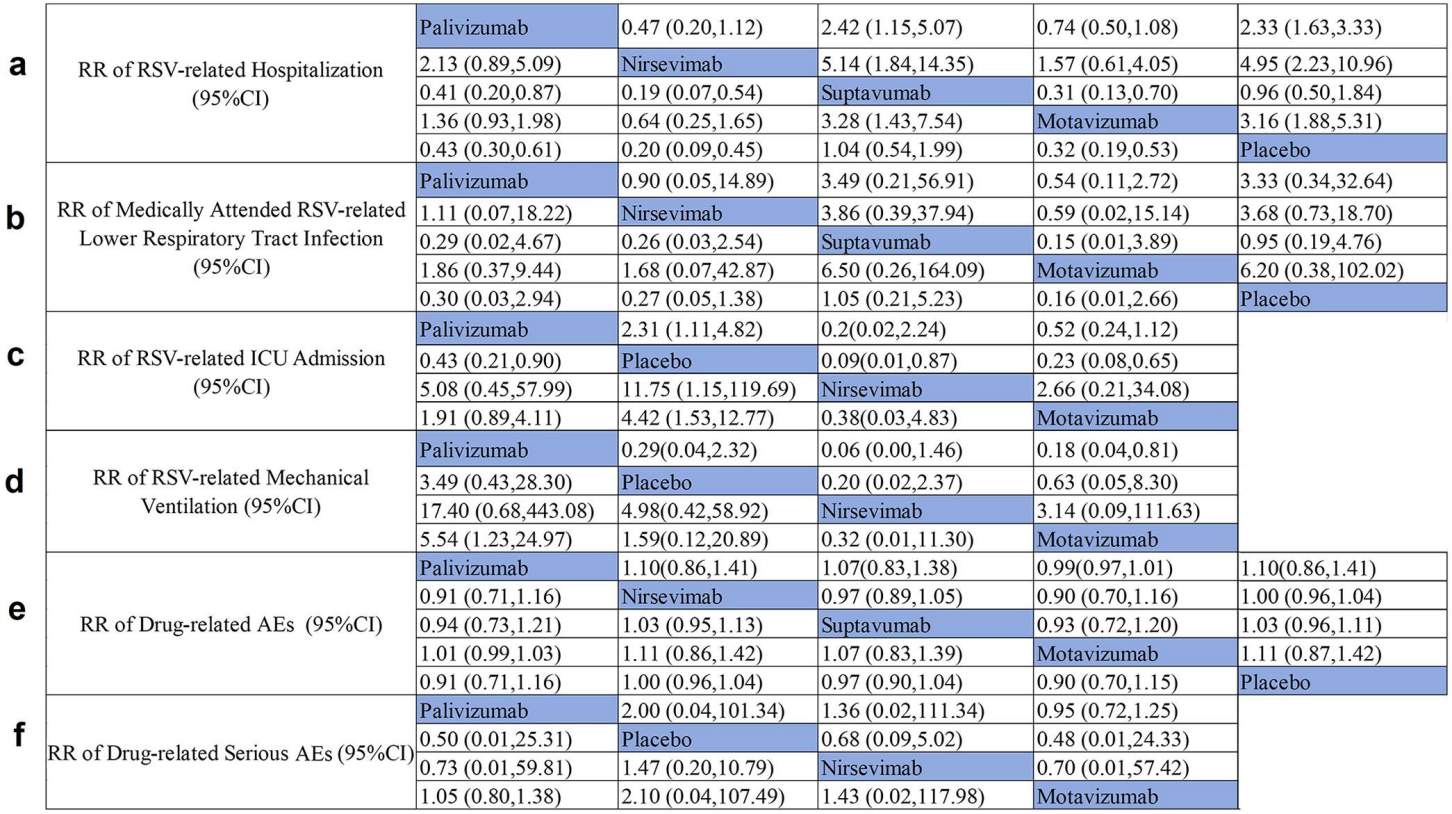
League table of pairwise comparisons among monoclonal antibody regimens for efficacy and safety outcomes against RSV disease in premature infants. This league table summarizes the results of network meta-analysis for six clinically relevant outcomes: **(a)** RSV-related hospitalization, **(b)** medically attended RSV-related lower respiratory tract infection, **(c)** RSV-related ICU admission, **(d)** RSV-related mechanical ventilation, **(e)** drug-related adverse events (AEs), and **(f)** drug-related serious adverse events (SAEs). The league table is structured as a square matrix with treatments listed on both rows and columns. League table cells show RR of column treatment/row treatment; RR < 1 favors the column treatment.

**Figure 4:**
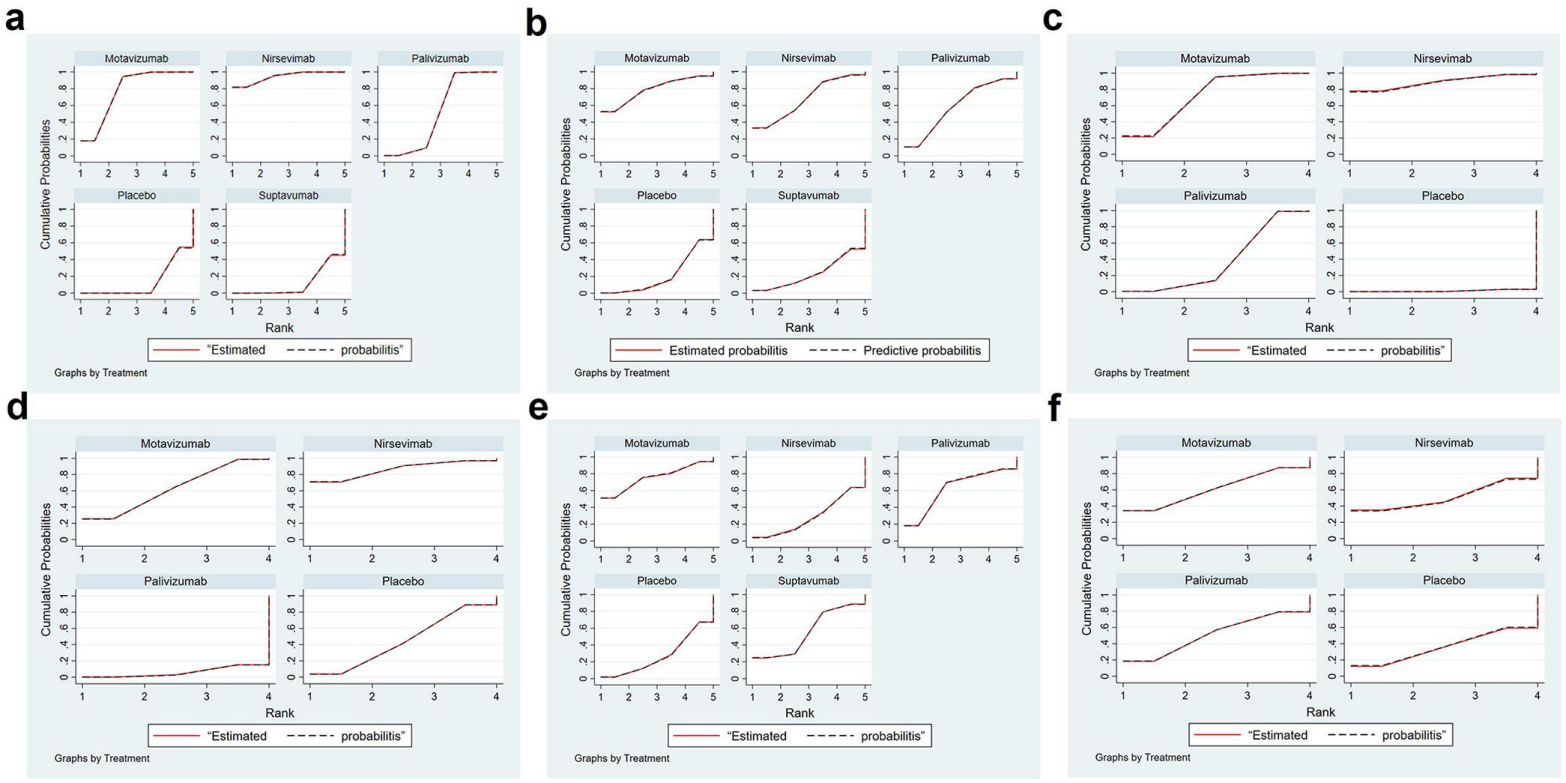
SUCRA plot of cumulative ranking probabilities for monoclonal antibodies against RSV disease in premature infants. Each panel displays the cumulative ranking probabilities for a specific outcome: **(a)** RSV-related hospitalization, **(b)** medically attended RSV-related lower respiratory tract infection, **(c)** RSV-related ICU admission, **(d)** RSV-related mechanical ventilation, **(e)** drug-related adverse events (AEs), and **(f)** drug-related serious adverse events (SAEs). Each curve represents the cumulative probability of a given intervention being ranked at each position (from best to worst) based on the network meta-analysis results. The *x*-axis shows the rank order (1 = best, *N* = worst), and the *y*-axis shows the cumulative probability. The surface under the cumulative ranking curve (SUCRA) value for each intervention is proportional to the area under its curve; a higher SUCRA value (closer to 1) indicates a better ranking.

However, no significant difference was observed between suptavumab and placebo (RR = 1.04, 95% CI: 0.54–1.99) (Figure 3a and Supplementary data S3 Figure S1a). Based on the SUCRA cumulative ranking probabilities, the comparative effectiveness ranking was as follows: nirsevimab (94.4%) > motavizumab (78.1%) > palivizumab (52.2%) > placebo (13.7%) > suptavumab (11.6%) (Figure 4a and Supplementary data S3 Table S2, Figure 2a).

#### 3.3.2 Medically attended RSV-related lower respiratory tract infection

Five studies 22–26 reported medically attended RSV-related lower respiratory tract infection outcomes (mAb group *n* = 4,401; placebo group *n* = 1,102). Compared with placebo, the RR was 0.27 (95% CI 0.05–1.38) for nirsevimab, 0.16 (95% CI 0.01–2.66) for motavizumab, and 0.30 (95% CI 0.03–2.94) for palivizumab. While the point estimates indicated a decreased risk of medically attended lower respiratory tract infection, none of the between-group differences attained statistical significance. No significant difference was observed for suptavumab vs. placebo (RR = 1.05, 95% CI 0.21–5.23) (Figure 3b and Supplementary data S3 Figure S1b). The SUCRA cumulative ranking probabilities results revealed the following ranking of comparative efficacy: motavizumab (79.0%) > nirsevimab (67.9%) > palivizumab (58.9%) > suptavumab (23.4%) > placebo (20.8%) (Figure 4b, Supplementary Data S3 Table S2, Figure S2b).

#### 3.3.3 ICU hospitalizations related to RSV infections

Four studies (21, 22, 25, 27) reported the ICU hospitalizations rate related to RSV infections in premature infants (mAb group *n* = 8,677; placebo group *n* = 1,002). Compared with placebo, the RRs for nirsevimab, motavizumab and palivizumab were 0.09 (95% CI 0.01–0.87), 0.23 (95% CI 0.08–0.65) and 0.43 (95% CI 0.21–0.90), respectively. All three treatments were statistically superior to placebo (Figure 3c and Supplementary data S3 Figure S1c). Based on the SUCRA cumulative ranking probabilities, the comparative effectiveness ranking was as follows: nirsevimab (89.1%) > motavizumab (72.2%) > palivizumab (37.6%) > placebo (1.0%) (Figure 4c, Supplementary data S3 Table S2, Figure S2c).

#### 3.3.4 Mechanical ventilation Use and deaths related to RSV infections

Four studies (21, 22, 25, 27) reported ICU hospitalizations related to RSV infections (mAb group *n* = 8,677; placebo group *n* = 1,002). Compared with placebo, nirsevimab, motavizumab, and palivizumab did not show significant advantages in mechanical ventilation use related to RSV infections in premature infants **(**Figure 3d and Supplementary data S3 Figure S1d), The SUCRA value of Nirsevimab was the highest with 86.2% (Figure 4d, Supplementary Data S3 Table S2,Figure S2d). Among the seven studies, only the IMpact-RSV Study Group (21) reported two RSV-related deaths in the intervention group. No other studies reported RSV-related deaths.

#### 3.3.5 Drug safety

All seven studies (21–27) reported adverse events, with 7,280 treatment-related adverse events occurring in the intervention group and 821 adverse events occurring in the placebo group. Compared with placebo, nirsevimab had an RR of 1.0 (95% CI: 0.96–1.04), motavizumab had an RR of 0.9 (95% CI: 0.70–1.15), and palivizumab had an RR of 0.91 (95% CI: 0.71, 1.16), and suptavumab had an RR of 0.97 (95% CI: 0.90, 1.04), all showing no significant difference (Figures 3e, 4e, Supplementary Data S3 Figures S1e, S2e). Four studies (21, 22, 25, 27) reported serious adverse events (SAEs), enrolling 8,677 infants in the mAb groups and 1,002 in the placebo group. A total of 1,104 SAEs occurred in the intervention groups and 81 in the placebo group. Compared with placebo, nirsevimab yielded an RR of 0.68 (95% CI: 0.09–5.02), motavizumab an RR of 0.48 (95% CI: 0.01–24.33), and palivizumab an RR of 0.50 (95% CI: 0.01–25.31), with no statistically significant differences (Figures 3f, 4f, Supplementary data S3 Figures 1f, 2f). Overall, mAbs did not significantly increase the risk of adverse events or SAEs compared with placebo. None of the seven included studies reported mortality related to drug side effects.

## 4 Discussion

Our network meta-analysis suggests that nirsevimab, motavizumab, and palivizumab may confer a protective benefit against RSV-related hospitalizations and ICU admissions in preterm infants compared with placebo, without a significant increase in adverse events. However, pairwise comparisons among the three monoclonal antibodies did not demonstrate statistically significant differences in either efficacy or safety. These findings are like those of a previous study (18) on all children.

Palivizumab, a human monoclonal antibody targeting the RSV fusion (F) glycoprotein, inhibits RSV replication (28), and is the most extensively investigated anti-RSV antibody. Approved in the United States in 1998, it is indicated for RSV prophylaxis in infants and children with underlying diseases. Due to its low cost-effectiveness (29), its application as a public health strategy for against RSV infection has been strictly limited nationwide since 2014. In line with earlier meta-analyses (30), this study confirmed that palivizumab lowers the rates of RSV-related hospitalization and ICU admission in preterm infants. Meanwhile, no significant decreases in RSV-attributable mortality or mechanical ventilation use were observed, in accordance with prior reports.

According to the SUCRA ranking, nirsevimab demonstrated the highest probability of being the most effective intervention for against RSV-related hospitalizations and ICU admissions in preterm infants. Nirsevimab is a long-acting recombinant human IgG1 monoclonal antibody engineered with YTE modifications in the Fc region to extend its serum half-life (31). This pharmacokinetic advantage enables a single dose to provide protection throughout the entire RSV season, offering a more convenient administration regimen compared with palivizumab and motavizumab, which require five monthly injections. Nirsevimab was first approved in the European Union and the United Kingdom on November 3 and November 7, 2022, respectively, for the against RSV infection in newborns and infants during their first RSV season. It was subsequently approved in China in 2024. Given that the seven studies included in this analysis were conducted exclusively in Europe and the United States, with a limited proportion of Asian participants, further studies are warranted to evaluate the efficacy and safety of nirsevimab in Asian populations. No significant differences were observed between the mAb and placebo groups in terms of RSV-related mechanical ventilation rates or RSV-related mortality, which may reflect the low incidence of these severe outcomes in the included studies. Regarding safety, no significant differences were found among the different mAbs compared with placebo, particularly for serious adverse events. However, it should be noted that motavizumab has been associated with significant hypersensitivity reactions, and the American Academy of Pediatrics does not recommend its clinical use. Furthermore, suptavumab failed to demonstrate efficacy in against RSV-associated hospitalizations or outpatient lower respiratory tract infections in children, resulting in trial termination. Additional studies are needed to confirm these findings, particularly regarding long-term safety and cost-effectiveness.

This study has several notable strengths. First, although prior meta-analyses have systematically reviewed the efficacy and safety of RSV monoclonal antibodies against RSV infection in infants and children broadly, none have specifically focused on preterm infants. To our knowledge, this is the first network meta-analysis to evaluate RSV monoclonal antibodies exclusively in the preterm infant population. Second, this review incorporated four monoclonal antibodies and included randomized controlled trials with a substantial total sample size (*n* = 11,319), enabling a comprehensive comparative analysis with adequate statistical power.

This study has several limitations. First, in the absence of head-to-head randomized comparisons, our network meta-analysis relied primarily on indirect evidence. Second, three of the seven included RCTs did not adequately describe allocation concealment, which may introduce selection bias. Third, all seven studies were conducted in high-income countries, with no data from low- or middle-income countries, limiting the generalizability of our findings to these settings. Fourth, although all studies provided detailed data on RSV-related hospitalizations, not all studies reported outcomes on lower respiratory tract infections, disease severity, or respiratory support requirements, precluding detailed subgroup analyses for these endpoints. Fifth, given the small number of included studies, publication bias could not be formally assessed, and study quality was evaluated using the risk of bias tool alone. Future studies should aim to increase sample sizes, incorporate additional monoclonal antibody trials, and include populations from diverse geographic and socioeconomic regions.

## 5 Conclusion

In conclusion, palivizumab, nirsevimab, and motoravizumab have shown positive effects at reducing RSV-related hospitalization and ICU admission rates in premature infants. Among these, nirsevimab has demonstrated the greatest potential. Suptavumab did not show any positive effects compared to the placebo. No significant effects on the reduction of RSV-related mechanical ventilation or mortality were observed.

## Data Availability

The original contributions presented in the study are included in the article/Supplementary Material, further inquiries can be directed to the corresponding author.
