# Supplementary material for: Efficacy and safety of monoclonal antibodies against respiratory syncytial virus disease in premature infants: a systematic review and network meta-analysis
Source: Front Pediatr. 2026 Jul 2;14:1775795. doi: 10.3389/fped.2026.1775795 (PMC13372888; doi:10.3389/fped.2026.1775795)
Supplement: Supplementary file 1 [file Datasheet1.docx]

**Supplementary data 3**

**sTable 1 Evidence evaluation by using ROB**

| **study** | **Randomization process generated** | **Deviations from the intended intervention** | **Missing data** | **Measurement of the outcome** | **Selection of the reported results** | **Overall** | **Quality rating** |
| --- | --- | --- | --- | --- | --- | --- | --- |
| The IMpact-RSV Study Group 1998[18] | Low | Low | Low | Low | Low | Low | high-quality |
| Griffin, M. P. 2020[19] | Low | Low | Low | Low | Low | Low | high-quality |
| Blanken, M. O. 2013[20] | Low | Low | Low | Low | Low | Low | high-quality |
| Simões, E. A. F. 2021[21] | Low | Low | Low | Low | Low | Low | high-quality |
| Carbonell-Estrany, X. 2010[22] | Low | Low | Low | Low | Some concerns | Some concerns | moderate-quality |
| Subramanian KN 1998[23] | Low | Low | Low | Low | Low | Low | high-quality |
| Domachowske JB 2018[24] | Low | Low | Low | Low | Low | Low | high-quality |

**sTable2：SUCRA cumulative rate tables for monoclonal antibodies against RSV disease in premature infants**

| Outcomes | Treatment | SUCRA(%) | PrBest(%) | MeanRank |
| --- | --- | --- | --- | --- |
| Sucra cumulative plot of RSV-related-Hospitalization | Palivizumab | 52.2 | 0.2 | 2.9 |
|  | Nirsevimab | 94.4 | 81.9 | 1.2 |
|  | Suptavumab | 11.6 | 0 | 4.5 |
|  | Motavizumab | 78.1 | 17.9 | 1.9 |
|  | Placebo | 13.7 | 0 | 4.5 |
| Sucra cumulative plot of Medically Attended RSV-related Lower Respiratory Tract Infection | Palivizumab | 58.9 | 10.4 | 2.6 |
|  | Nirsevimab | 67.9 | 32.7 | 2.3 |
|  | Suptavumab | 23.4 | 3.1 | 4.1 |
|  | Motavizumab | 79.0 | 53.5 | 1.8 |
|  | Placebo | 20.8 | 0.2 | 4.2 |
| Sucra cumulative plot of RSV-related-ICU Admission | Palivizumab | 37.6 | 0.4 | 2.9 |
|  | Placebo | 1 | 0 | 4 |
|  | Nirsevimab | 89.1 | 78 | 1.3 |
|  | Motavizumab | 72.2 | 21.6 | 1.8 |
| Sucra cumulative plot of RSV-related-Mechanical Ventilation Use | Palivizumab | 6 | 0 | 3.8 |
|  | Placebo | 44.8 | 3.7 | 2.7 |
|  | Nirsevimab | 86.2 | 70.7 | 1.4 |
|  | Motavizumab | 63 | 25.5 | 2.1 |
| Sucra cumulative plot of Drug-related Adverse Events | Palivizumab | 62.6 | 18.3 | 2.5 |
|  | Nirsevimab | 29.1 | 4.4 | 3.8 |
|  | Suptavumab | 55.5 | 24.5 | 2.8 |
|  | Motavizumab | 75.3 | 51 | 2 |
|  | Placebo | 27.5 | 1.8 | 3.9 |
| Sucra cumulative plot of Drug-related Serious Adverse Events | Palivizumab | 51.5 | 18.4 | 2.5 |
|  | Placebo | 35.7 | 12.1 | 2.9 |
|  | Nirsevimab | 51.5 | 35.2 | 2.5 |
|  | Motavizumab | 61.2 | 34.3 | 2.2 |

SUCRA: surface under the cumulative ranking curve (higher=better): PrBest: probability of being the best treatment (higher = better): MeanRank: average rank position (lower = better).

**sFig1. Forest plots of pairwise comparisons for efficacy and safety outcomes against RSV disease in premature infants: all monoclonal antibody interventions compared with placebo**


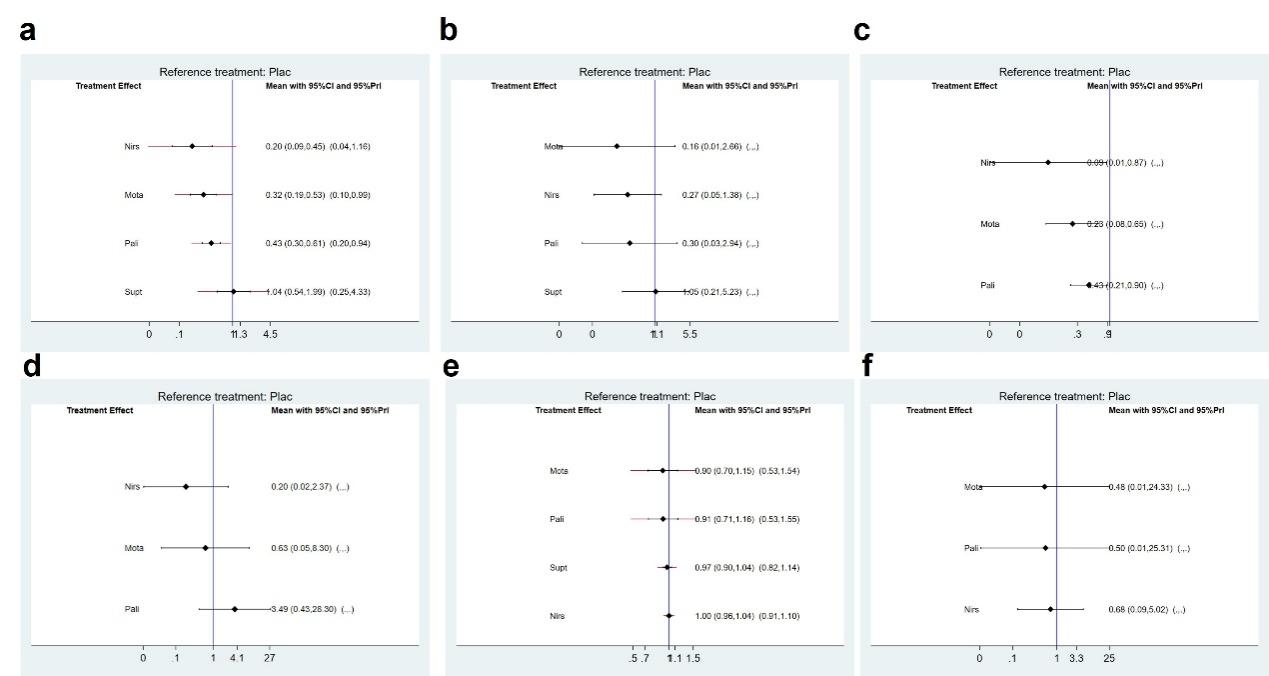


(a) Forest Plots of mAb prevention of RSV-related-Hospitalization compared with placebo;(b) Forest Plots for medically attended RSV‑related lower respiratory tract infection compared with placebo; (c) Forest Plots for RSV‑related ICU admission compared with placebo; (d) Forest Plots for RSV‑related mechanical ventilation compared with placebo; (e) Forest Plots of drug-related adverse events compared with placebo; (f) Forest Plots for Drug-related Serious Adverse Events compared with placebo. Pali: Palivizumab; Nirs: Nirsevimab; Supt: Suptavumab; Mota: Motavizumab; plac: Placebo

**sFig2. Rank probability plots for monoclonal antibody interventions against RSV in premature infants: the probability that each treatment is ranked at a particular position is shown in each panel**


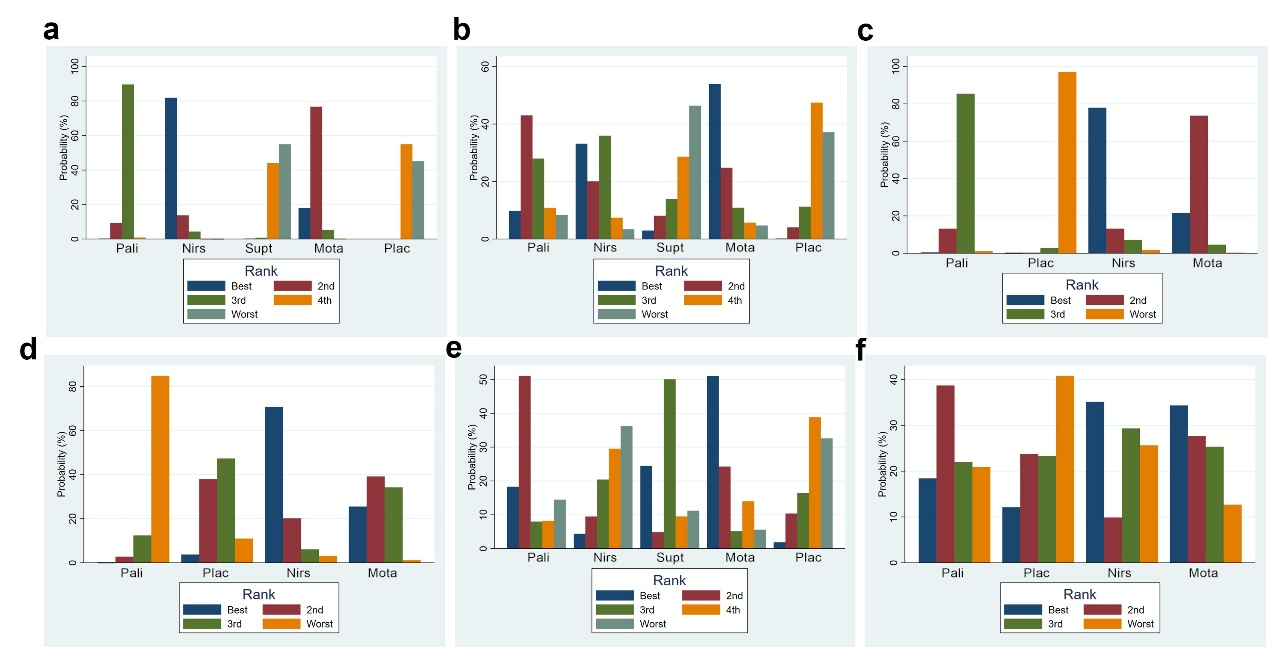


1. RANK probability plots for RSV-related-Hospitalization; (b) RANK probability plots for medically attended RSV-related lower respiratory tract infection (c) RANK probability plots for RSV-related-ICU Admission; (d) RANK probability plots for RSV-related Mechanical Ventilation Use; (e) RANK probability plots for Drug-related Adverse Events; (f) RANK probability plots for Drug-related Serious Adverse Events. Pali: Palivizumab; Nirs: Nirsevimab; Supt: Suptavumab; Mota: Motavizumab; plac: Placebo; X-axis: All study interventions; Y- axis: Rank probability (0% to 100%).
