# Supplementary material for: Efficacy and safety of monoclonal antibodies against respiratory syncytial virus disease in premature infants: a systematic review and network meta-analysis
Source: Front Pediatr. 2026 Jul 2;14:1775795. doi: 10.3389/fped.2026.1775795 (PMC13372888; doi:10.3389/fped.2026.1775795)
Supplement: Supplementary file 2 [file Table1.doc]

**Embase search strategy**

'human respiratory syncytial virus'/exp OR 'Respiratory Syncytial Virus':ti,ab,kw OR 'Syncytial Virus, Respiratory':ti,ab,kw OR 'Virus, Respiratory Syncytial':ti,ab,kw OR 'RSV Respiratory Syncytial Virus':ti,ab,kw OR 'Orthopneumovirus':ti,ab,kw OR 'Orthopneumoviruses':ti,ab,kw OR 'Chimpanzee Coryza Agent':ti,ab,kw OR 'Chimpanzee Coryza Agents':ti,ab,kw OR 'Coryza Agent, Chimpanzee':ti,ab,kw OR 'RSV':ti,ab,kw

'nirsevimab'/exp OR 'beyfortus':ti,ab,kw OR 'MED-18897':ti,ab,kw OR 'MED18897':ti,ab,kw

'palivizumab'/exp OR 'MEDI 493':ti,ab,kw OR 'Monoclonal Antibody MEDI-493':ti,ab,kw OR 'Monoclonal Antibody MEDI 493':ti,ab,kw OR 'Monoclonal Antibody MEDI493':ti,ab,kw OR 'MEDI-493':ti,ab,kw OR 'MEDI493':ti,ab,kw OR 'Synagis':ti,ab,kw

'motavizumab'/exp OR 'Numax':ti,ab,kw OR 'MEDI-524':ti,ab,kw

'Suptavumab'/exp OR

'Clesrovimab'/exp

'prematurity'/exp OR 'premature birth':ti,ab,kw OR 'birth, premature':ti,ab,kw OR 'births, premature':ti,ab,kw OR 'premature births':ti,ab,kw OR 'preterm birth':ti,ab,kw OR 'birth, preterm':ti,ab,kw OR 'births, preterm':ti,ab,kw OR 'preterm births':ti,ab,kw OR 'infant, premature':ti,ab,kw OR 'infants, premature':ti,ab,kw OR 'premature infant':ti,ab,kw OR 'preterm infants':ti,ab,kw OR 'infant, preterm':ti,ab,kw OR 'infants, preterm':ti,ab,kw OR 'preterm infant':ti,ab,kw OR 'premature infants':ti,ab,kw OR 'neonatal prematurity':ti,ab,kw OR 'prematurity, neonatal':ti,ab,kw OR 'obstetric labor, premature':ti,ab,kw OR 'labor, premature obstetric':ti,ab,kw OR 'labor, premature':ti,ab,kw OR 'premature labor':ti,ab,kw OR 'premature obstetric labor':ti,ab,kw OR 'preterm labor':ti,ab,kw OR 'labor, preterm':ti,ab,kw

**Pubmed search strategy**

((((((((((Obstetric Labor, Premature[Title/Abstract]) OR (Labor, Premature Obstetric[Title/Abstract])) OR (Premature Labor[Title/Abstract])) OR (Labor, Premature[Title/Abstract])) OR (Premature Obstetric Labor[Title/Abstract])) OR (Preterm Labor[Title/Abstract])) OR (Labor, Preterm[Title/Abstract])) OR ((((((((Premature Birth[Title/Abstract]) OR (Birth, Premature[Title/Abstract])) OR (Births, Premature[Title/Abstract])) OR (Premature Births[Title/Abstract])) OR (Preterm Birth[Title/Abstract])) OR (Birth, Preterm[Title/Abstract])) OR (Births, Preterm[Title/Abstract])) OR (Preterm Births[Title/Abstract]))) OR ((((((((((Infant, Premature[Title/Abstract]) OR (infants, Premature[Title/Abstract])) OR (Premature Infant[Title/Abstract])) OR (Preterm Infants[Title/Abstract])) OR (Infant, Preterm[Title/Abstract])) OR (Infants, Preterm[Title/Abstract])) OR (Preterm Infant[Title/Abstract])) OR (Premature Infants[Title/Abstract])) OR (Neonatal Prematurity[Title/Abstract])) OR (Prematurity, Neonatal[Title/Abstract]))) AND (((((((nirsevimab[Title/Abstract]) OR (beyfortus[Title/Abstract])) OR (MED-18897[Title/Abstract])) OR (MED18897[Title/Abstract])) OR ((((((((palivizumab[Title/Abstract]) OR (MEDI 493[Title/Abstract])) OR (Monoclonal Antibody MEDI-493[Title/Abstract])) OR (Monoclonal Antibody MEDI 493[Title/Abstract])) OR (Monoclonal Antibody MEDI493[Title/Abstract])) OR (MEDI-493[Title/Abstract])) OR (MEDI493[Title/Abstract])) OR (Synagis[Title/Abstract]))) OR (((motavizumab[Title/Abstract]) OR (Numax[Title/Abstract])) OR (MEDI-524[Title/Abstract]))) OR (Suptavumab[Title/Abstract]))) OR (Clesrovimab[Title/Abstract])))) AND ((((((((((((Respiratory Syncytial Virus[MeSH Terms]) OR (Respiratory Syncytial Virus[Title/Abstract])) OR (Respiratory Syncytial Virus[Title/Abstract])) OR (Syncytial Virus, Respiratory[Title/Abstract])) OR (Virus, Respiratory Syncytial[Title/Abstract])) OR (RSV Respiratory Syncytial Virus[Title/Abstract])) OR (Orthopneumovirus[Title/Abstract])) OR (Orthopneumoviruses[Title/Abstract])) OR (Chimpanzee Coryza Agent[Title/Abstract])) OR (Chimpanzee Coryza Agents[Title/Abstract])) OR (Coryza Agent, Chimpanzee[Title/Abstract])) OR (RSV[Title/Abstract]))

**Cochrane search strategy**

Search Name:

Date Run: 24/12/2025 03:26:42

Comment:

ID Search Hits

#1 Respiratory Syncytial Virus 1554

#2 Respiratory Syncytial Viruses 339

#3 MeSH descriptor: [Respiratory Syncytial Viruses] explode all trees 328

#4 Syncytial Virus, Respiratory 1554

#5 Virus, Respiratory Syncytial 1554

#6 RSV Respiratory Syncytial Virus 1159

#7 Orthopneumovirus 3

#8 Orthopneumoviruses 0

#9 Chimpanzee Coryza Agent 2

#10 Chimpanzee Coryza Agents 2

#11 Coryza Agent, Chimpanzee 2

#12 RSV 1452

#13 #1 or #2 or #3 or #4 or #5 or #6 or #7 or #8 or #9 or #10 or #11 or #12 1860

#14 nirsevimab 46

#15 beyfortus 4

#16 MED-18897 0

#17 MED18897 0

#18 #15 or #16 or #17 or #14 46

#19 Palivizumab 178

#20 MEDI 493 26

#21 Monoclonal Antibody MEDI-493 12

#22 Monoclonal Antibody MEDI493 0

#23 Monoclonal Antibody MEDI 493 12

#24 MEDI-493 14

#25 MEDI493 0

#26 Synagis 42

#27 #20 or #21 or #22 or #23 or #24 or #25 or #26 or #19 194

#28 motavizumab 29

#29 Numax 5

#30 MEDI-524 18

#31 #29 or #30 or #28 34

#32 Suptavumab 3

#33 Clesrovimab 16

#34 #18 or #27 or #31 or #32 or #33 253

#35 MeSH descriptor: [Infant, Premature] explode all trees 5857

#36 infants, Premature 10395

#37 Premature Infant 11157

#38 Preterm Infants 11928

#39 Infant, Preterm 10987

#40 Infants, Preterm 11928

#41 Preterm Infant 10987

#42 Premature Infants 10395

#43 Neonatal Prematurity 5253

#44 Prematurity, Neonatal 5253

#45 MeSH descriptor: [Premature Birth] explode all trees 2604

#46 Birth, Premature 10334

#47 Births, Premature 902

#48 Premature Birth 10334

#49 Preterm Birth 11537

#50 Birth, Preterm 11537

#51 Births, Preterm 1172

#52 Preterm Births 1172

#53 MeSH descriptor: [Obstetric Labor, Premature] explode all trees 3443

#54 Labor, Premature Obstetric 1584

#55 Labor, Premature 3547

#56 Premature Labor 3547

#57 Premature Obstetric Labor 1584

#58 Preterm Labor 3569

#59 Labor, Preterm 3569

#60 #35 or #36 or #37 or #38 or #39 or #40 or #41 or #42 or #43 or #44 or #45 or #46 or #47 or #48 or #49 or #50 or #51 or #52 or #53 or #54 or #45 or #46 or #57 or #58 or #59 24781

#61 #60 and #34 and #13 98 (reviews n =7）

Pubmed 371

Embase 929

Chocrane 91
